# Validity and reliability of the self-rated fall risk questionnaire in older adults with osteoporosis

**DOI:** 10.1186/s12891-020-03788-z

**Published:** 2020-11-18

**Authors:** Nitchanant Kitcharanant, Ekasame Vanitcharoenkul, Aasis Unnanuntana

**Affiliations:** Department of Orthopedic Surgery, Faculty of Medicine Siriraj Hospital, Mahidol University, 2 Wanglang Road, Bangkoknoi, Bangkok, 10700 Thailand

**Keywords:** Validity, Reliability, Self-rated fall risk questionnaire, Older adults, Osteoporosis

## Abstract

**Background:**

Several risk assessments have been developed to evaluate fall risk in older adults, but it has not been conclusively established which of these tools is most effective for assessing fall risk in this vulnerable population. Recently, the U.S. Centers for Disease Control and Prevention (CDC) developed the self-rated Fall Risk Questionnaire (self-rated FRQ), a 12-item questionnaire designed to screen older adults who are at risk of falling and has been widely used in many centers. This study aimed to determine the validity and reliability of the self-rated FRQ in older adults with osteoporosis.

**Methods:**

This prospective study was conducted at the Department of Orthopedic Surgery, Faculty of Medicine Siriraj Hospital, Mahidol University, Bangkok, Thailand from December 2019 to March 2020. Sixty-eight men or postmenopausal women aged > 65 years who were diagnosed with osteoporosis either by bone mineral density T-score or by occurrence of fragility fracture were evaluated with the self-rated FRQ, the Thai falls risk assessment test (Thai-FRAT), the timed get-up-and-go test (TUG test), the Berg Balance Scale (BBS), and the 5 times sit-to-stand test (5TSTS test). Validity of the self-rated FRQ was assessed by evaluating the correlations (*r*) between the self-rated FRQ score and the scores from the other four assessments. Reliability of the self-rated FRQ was evaluated by measuring test-retest reliability and internal consistency.

**Results:**

The self-rated FRQ was moderately strongly correlated with the BBS, TUG test, and 5TSTS test (*r* = 0.535 to 0.690; *p* < 0.001), and fairly correlated with the Thai-FRAT (*r* = 0.487; *p* < 0.001). Test-retest reliability of the self-rated FRQ was high, with a Kappa of 1. Internal consistency of the self-rated FRQ was excellent (Cronbach’s alpha: 0.936).

**Conclusions:**

The self-rated FRQ was found to be a valid and reliable tool for evaluating fall risk in older adults with osteoporosis. Since assessment of fall risk requires a multifaceted measurement tool, the self-rated FRQ is an appropriate tool that can be integrated into the fall risk assessment algorithm in older adults with osteoporosis.

**Supplementary Information:**

The online version contains supplementary material available at 10.1186/s12891-020-03788-z.

## Introduction

Osteoporosis is a silent and progressive skeletal disorder that is characterized by low bone mineral density and deterioration of bone micro-architecture [1]. As more societies around the world become aging or aged societies, the incidence of osteoporosis and its most serious complication, fragility fracture, are also increasing [2]. Osteoporotic patients are known to have decreased muscle strength, postural changes, and poor body balance, which collectively can lead to an increased risk of falls [3, 4]. Fragility fracture usually occurs after a simple fall, and subsequently leading to increased disability, morbidity, and mortality [5]. A previous study reported a 25% mortality rate among fall-related hip fracture patients aged over 65 years [6]. Among those who have already sustained a fragility hip fracture, there is a high probability of a second fall and a second fracture [7]. Therefore, a reliable tool for assessing fall risk in older adults with osteoporosis is urgently needed.

Although a number of risk assessment tools have been developed to evaluate fall risk in older adult populations [8, 9], it remains controversial which of these tools is the optimal assessment for fall risk. Some fall risk assessment tools ask the patient to perform specific tasks, such as the timed get-up-and-go test (TUG test) and the Berg Balance Scale (BBS). However, these performance-based tests are difficult to routinely administer in an outpatient setting. Recently, the U.S. Centers for Disease Control and Prevention (CDC) developed the self-rated Fall Risk Questionnaire (self-rated FRQ), a 12-item questionnaire designed to screen older adults who are at risk of falling, and is a component of the STEADI toolkit, which provides patient and health care provider fall prevention education materials [10].

The self-rated FRQ has been shown to have good concurrent validity [11], and has been widely used in many centers. Although a Thai language translation of the self-rated FRQ has been validated, the study only included community-dwelling older adults. No such validation of this tool has been performed in older Thai adults with osteoporosis, therefore, the objectives of this study were to develop the Thai self-rated FRQ, and to test its validity and reliability in older Thai adults with osteoporosis.

## Methods

The study protocol and consent form were both approved by the Siriraj Institutional Review Board (SIRB) of the Faculty of Medicine Siriraj Hospital, Mahidol University, Bangkok, Thailand (COA no. Si 749/2019). This study was conducted in accordance with the principles described in the Declaration of Helsinki and all of its later amendments. The original English language version of the self-rated FRQ was translated into Thai language using a forward-backward translation technique according to guidelines proposed by Beaton et al. [12]. This process involves two translations of the self-rated FRQ from English to Thai, one by a professional English translator and one by a bilingual physician. The two translations were then reviewed and adapted into one version. Backward translation from Thai into English was performed by a local professional translator who had no access to or awareness about the original self-rated FRQ. The backward version was then compared to the original English self-rated FRQ to identify any discrepancies. This process resulted in the development of the final version. Afterwards, a group of experts in falls in older adults with osteoporosis that consisted of 2 orthopedic surgeons, 1 physiatrist, 1 geriatrician, and 1 fracture liaison service nurse were asked to evaluate the content validity of the self-rated FRQ. Each expert was asked to rate each item on the self-rated FRQ with a score of 1 (agree), 0 (unsure/ unclear), or − 1 (disagree) to evaluate the content validity. The average score of each questionnaire item was then used to calculate the index of item-objective congruence. We affirmed the understandability of the questions by exposing 15 randomly selected osteoporotic patients to the self-rated FRQ as the final step of the translation protocol.

### Validation

#### Patients

The authors prospectively enrolled patients from the outpatient orthopedic unit of the Department of Orthopedic Surgery, Faculty of Medicine Siriraj Hospital, Mahidol University, Bangkok, Thailand from December 2019 to March 2020. We included men and postmenopausal women aged more than 65 years who were diagnosed with osteoporosis by bone mineral density (BMD) T-score ≤ − 2.5 or by history of low-energy hip or vertebral compression fracture (VCF). Patients with severe cognitive and/or neurological impairment (e.g., dementia), deafness, or severe cardiopulmonary diseases for whom it would not be safe to undergo performance-based testing were excluded. All included patients were evaluated by the same investigator (NK). Data, including age, gender, body mass index, Charlson comorbidity index, type of surgery, pre-injury walking status, and living condition, were collected and recorded. Written informed consent was obtained from each patient who participated in this study.

#### Outcome measurement tools

Each participant was asked to complete the self-rated FRQ and the Thai Falls Risk Assessment Test (Thai-FRAT). Participants were also asked to perform the 3 following performance-based tests: BBS, TUG test, and the 5 times sit-to-stand test (5TSTS test). To determine the test-retest reliability of the self-rated FRQ, a second copy of the self-rated FRQ was provided to the first 30 patients in a stamped, self-addressed envelope. One week later, those patients were reminded by telephone to complete the provided second copy of the self-rated FRQ, and to return it by Thailand Post to the research team.

### Self-rated fall risk questionnaire (self-rated FRQ)

The self-rated FRQ, which is the fall risk screening component of the STEADI algorithm [13], comprises 12 questions specific to individual physical functional performance and different fall risk factors. Each question can be scored as 0 or 1, or 0 or 2 depending on the question, and the total possible score is 14. A higher score indicates a higher risk of falling. The original version of the self-rated FRQ was shown to have good validity and reliability for evaluating older adult patients who are at high risk of falling with a cutoff value of 4 [11, 14]. If a patient scored 4 points or more, their fall risk was considered to be increased.

### Thai falls risk assessment test (Thai-FRAT)

Thai-FRAT is a fall risk assessment tool that was developed by a group of Thai investigators in 2008 to assess fall risk in Thai community-dwelling older adults. It consists of 6 items that identify fall risk factors, including gender, visual impairment, balance impairment, medication use, history of falls, and type of residence. The scoring range varies according to the item for a possible total score of 11. Thai-FRAT has good validity and reliability for identifying older adult patients who are at high risk for falling with a cutoff value of 4 [15]. If a patient scored 4 points or more, their fall risk was considered to be increased.

### Berg balance scale (BBS)

BBS is a performance-based test that asks patients to perform 14 tasks that are used to assess patient balance. Each task has a 5-point scale with a scoring range from 0 to 4 for a maximum total score of 56. A lower score indicates higher risk for falls. BBS has good validity and reliability for identifying older adult patients who are at high risk for falls with a cutoff value of 45 [16]. If a patient scores less than 45 points, their fall risk was regarded as being increased.

### Timed get-up-and-go test (TUG test)

Patients were asked to stand up from a high-seated chair, walk to a mark 3 m away at a comfortable pace, and then return to a sitting position [17]. Patients were allowed to use their arms when getting up from or sitting down in the seat. Patients were asked to perform this task three times, and the average time to complete the test was calculated and recorded. TUG test was reported to be a reliable tool for identifying risk of falls among older adult patients [18]. A threshold of 12 s was used as the cutoff value for detecting older adult patients at high risk for falling [18]. If patients took 12 s or longer to perform the TUG test, they were considered to be at possible risk of falling.

### 5 times sit-to-stand test (5TSTS test)

The 5TSTS test was conducted by asking patients to sit upright in an armless chair with a seat height of 43 cm with their arms crossed across their chest. The evaluator started timing after speaking the word “go”, after which patients stand up from a chair (without pushing off) and sit back down 5 times as quickly as possible. Timing stopped when the patients’ buttocks reached the chair after completing the fifth stand up-sit down cycle [19]. The current evidence demonstrates the efficacy of the 5TSTS for determining risk of falls in older adult patients with a cutoff value of 15 s [20]. If patients took longer than 15 s to perform the 5TSTS test, they were classified as being at higher risk of falling.

### Data analysis

Data analyses were performed using SPSS Statistics version 18 (SPSS, Inc., Chicago, IL, USA). Kolmogorov-Smirnov test was used to assess the distribution of data. Content validity of the self-rated FRQ was determined using the index of item-objective congruence. Construct validity was evaluated by comparing scores from the self-rated FRQ with scores from the BBS, Thai-FRAT, TUG, and 5TSTS tests. Construct validity was determined using chi-square test and Spearman’s rank correlation coefficient. A Spearman’s rank correlation coefficient (*r*) of < 0.3 was considered poor; 0.3 to 0.5, fair; > 0.5 to 0.8, moderately strong; and, > 0.8, very strong [21]. Since osteoporosis can be diagnosed using BMD T-score or by the occurrence of a fragility fracture, osteoporotic patient with a history of fragility fracture may have different characteristics when compared to those without fracture. Therefore, we have further evaluated construct validity in these two subgroup populations.

Test-retest reliability of the self-rated FRQ was also tested using Kappa statistics. A Kappa value of 0.1 to 0.2 was considered slight; 0.21 to 0.4, fair; 0.41 to 0.6, moderate; 0.61 to 0.8, substantial; and > 0.8, almost perfect [21, 22]. Internal consistency of the self-rated FRQ was assessed using Cronbach’s alpha. A Cronbach’s alpha within the range of 0.8 to 0.9 was considered good, and a value ≥0.9 was considered excellent [23]. The distribution of scores was calculated to evaluate for ceiling and floor effect. A ceiling or floor effect was considered to exist if > 15% of subjects achieved the lowest or highest possible score [23].

The sample size was estimated based on correlation between two tests. We assumed that the self-rated FRQ had very good correlation with BBS (|*r*|, absolute *r* = 0.75). Using a 2-sided type I error of 0.05 and 90% power to test H_0_: *ρ* = 0.5 versus H_1_: *ρ* = 0.75, a sample of 62 subjects was required. To compensate for a 10% attrition rate for any reason, the sample size was increased to 68.

## Results

### Translation and adaptation

There was no discrepancy between the backward-translated English version of the self-rated FRQ and the original version. The self-rated FRQ was then presented to older adult osteoporotic patients to evaluate its understandability. From 15 randomly selected osteoporotic patients, none reported any difficulty in understanding the questions. No missing data were observed, and no patients offered any suggestion for improvement of the questionnaire. All 12 items on the self-rated FRQ had good content validity. One item (I often feel sad or depressed) had an index of item-objective congruence of 0.8, whereas the other 11 items had an index of item-objective congruence of 1.0. This version of the self-rated FRQ was evaluated for validation and reliability.

### Validation and reliability

Eighty patients were screened during the study period. Twelve patients were excluded for the following reasons: 8 patients with severe cognitive and/or neurologic impairment, 3 patients with deafness, and 1 patient with severe cardiopulmonary disease that was considered to be at risk of sustaining harm during the performance-based tests. The remaining 68 patients were included. The mean age of study participants was 73.8 years (range: 65–88 years), and most subjects were female (91.2%) (Table 1). Fifty-three patients (77.9%) had a Charlson comorbidity index ≥3. Thirty-four patients (50%) had history of low-energy hip or vertebral compression fracture which occurred 2 or more years earlier. Those patients were assigned to the fracture group, whereas patients without a history of fracture were allocated to the non-fracture group. Fifty-nine patients (86.8%) walked without a gait aid, and the majority (94.1%) were considered community-dwelling older adults who could ambulate outdoors independently.

**Table 1:** Patient demographic and clinical characteristics

Patient characteristics	Total (***N*** = 68)	Fracture group (***n*** = 34)	Non-fracture group (***n*** = 34)	***p***-value
Age (years), mean ± SD	73.8 ± 6.2	75.9 ± 6.6	71.7 ± 5.1	***0.009***
Female gender, n (%)	62 (91.2%)	28 (82.4%)	34 (100.0%)	***0.025***
Body mass index (kg/m^2^), mean ± SD	24.0 ± 4.1	24.5 ± 4.4	23.5 ± 3.7	0.334
Charlson comorbidity index (CCI), n (%)				
2	15 (22.1%)	3 (8.8%)	12 (35.3%)	0.008
≥ 3	53 (77.9%)	31 (91.2%)	22(64.7%)	
Location of fracture, n(%)				
No fracture	34 (50.0%)	0 (0.0%)	34 (100.0%)	***< 0.001***
Vertebral compression fracture	23 (33.8%)	23 (67.6%)	0 (0.0%)	
Hip fracture	11 (16.2%)	11 (32.4%)	0 (0.0%)	
Ambulatory status, n(%)				
Household	4 (5.9%)	4 (11.8%)	0 (0.0%)	0.114
Community	64 (94.1%)	30 (88.2%)	34 (100.0%)	
Assisting device, n (%)				
Without assisting device	59 (86.8%)	26 (76.5%)	33 (97.1)	***0.027***
With assisting device	9 (13.2%)	8 (23.5%)	1 (2.9%)	
Bone mineral density (g/cm^2^), mean ± SD				
Lumbar spine	0.888 ± 0.177	0.989 ± 0.277	0.856 ± 0.118	0.150
Femoral neck	0.678 ± 0.106	0.672 ± 0.118	0.685 ± 0.095	0.636
Total hip	0.745 ± 0.118	0.742 ± 0.136	0.749 ± 0.098	0.801
Type of anti-osteoporotic drug, n (%)				
Oral bisphosphonate	44 (64.7%)	22 (64.7%)	22 (64.7%)	1.000
Intravenous bisphosphonate	6 (8.8%)	3 (8.8%)	3 (8.8%)	
Denosumab	18 (26.5%)	9 (26.5%)	9 (26.5%)	

A *p*-value< 0.05 indicates statistical significance (Student’s *t*-test)

*Abbreviation*: *SD* Standard deviation

The fracture group was significantly older (*p* = 0.009), had a significantly lower proportion of female patients (*p* = 0.025), and was significantly more likely to have a Charlson comorbidity index ≥3 (*p* = 0.008) compared to the non-fracture group. In addition, approximately one-quarter of fracture group patients used a gait aid compared to only 2.9% in the non-fracture group (*p* = 0.027). There was no significant difference in bone mineral density, ambulatory status, or type of anti-osteoporotic drugs between groups.

**Fig. 1:**
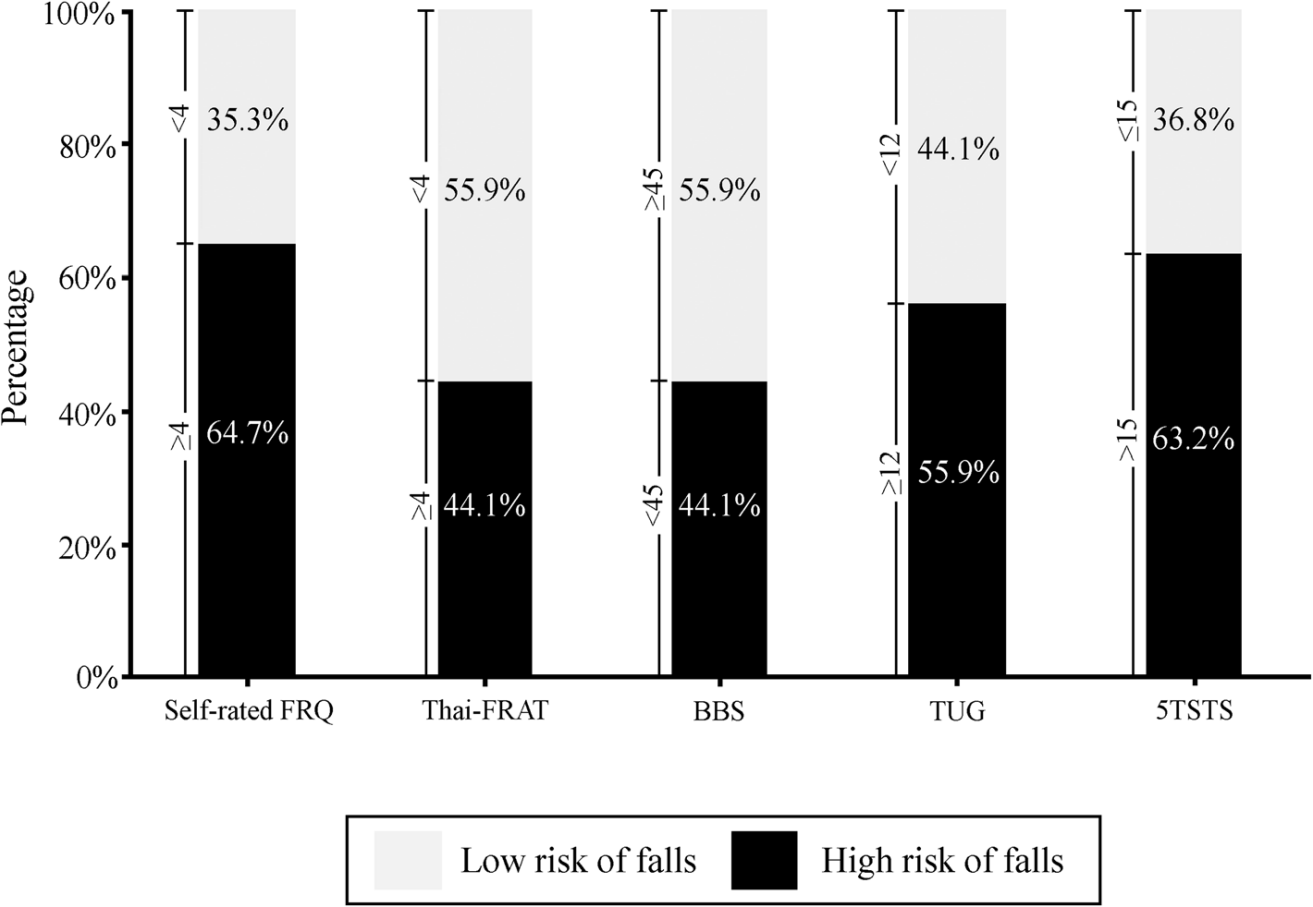
Percentage of patients classified as high-risk and low-risk of falls based on the cut-off value for each outcome measurement (Abbreviations: Self-rated FRQ, Self-rated Fall Risk Questionnaire; Thai-FRAT, Thai Falls Risk Assessment Test; BBS, Berg Balance Scale; TUG test, Timed get-up-and-go test; 5TSTS test, 5 times sit-to-stand test)

The self-rated FRQ score and other outcome measurement scores were found not to be normally distributed (Kolmogorov-Smirnov test, *p* < 0.05). Baseline outcome measurement scores compared between the fracture and non-fracture groups are shown in Table 2. The median self-rated FRQ and Thai-FRAT scores were 6 and 3 points, respectively. When we compared patient scores between the fracture and non-fracture groups, the median self-rated FRQ score was significantly higher in those with a history of fracture (7 and 5 points for the fracture and non-fracture groups, respectively; *p* = 0.009). The median Thai-FRAT scores for the fracture and non-fracture groups were 3 and 2.5 points, respectively. There was no difference in the Thai-FRAT scores between the fracture and non-fracture groups (*p* = 0.31). The median BBS score was 50. The median time used to complete the TUG and 5TSTS tests was 13 and 16.2 s, respectively. Similar to the self-rated FRQ, the scores of the BBS, TUG test, and 5TSTS test were all significantly different between the fracture and non-fracture groups (Table 2). Approximately 65% of patients were classified as being at high risk for falling based on the 4-point cutoff value for the self-rated FRQ (Fig. 1).

**Table 2:** Baseline patient scores for the 5 outcome measures compared between the fracture and non-fracture groups

Outcome measure	Total score Median (IQR)	Fracture group Median (IQR)	Non-fracture group Median (IQR)	***p-***value
Self-rated FRQ	6.0 (2.0, 8.0)	7.0 (3.0, 9.0)	5.0 (1.8, 7.0)	***0.009***
Thai-FRAT	3.0 (2.0, 4.0)	3.0 (2.0, 5.0)	2.5 (2.0, 4.0)	0.310
BBS	50.0 (43.0, 55.0)	44.0 (41.8, 52.3)	53.0 (44.0, 55.3)	***0.013***
TUG test	13.0 (10.6, 16.2)	13.9 (11.7, 21.7)	11.5 (10.4, 13.7)	***0.005***
5TSTS test	16.2 (13.9, 20.2)	18.8 (14.9, 25.5)	15.3 (12.9, 18.2)	***0.004***

A *p*-value< 0.05 indicates statistical significance (Mann-Whitney U test)

*Abbreviations***:** *IQR* Interquartile range, *Self-rated FRQ* Self-rated Fall Risk Questionnaire, *Thai-FRAT* Thai Falls Risk Assessment Test, *BBS* Berg Balance Scale, *TUG test* Timed get-up-and-go test, *5TSTS test* 5 times sit-to-stand test

Our analysis to evaluate for construct validity is presented in Table 3. The correlations between the score of the self-rated FRQ and all other outcome measurements were statistically significant (*p* < 0.001). A moderately strong correlation was observed between the self-rated FRQ and the BBS, TUG test, and 5TSTS test (|*r*| = 0.69, 0.535, and 0.566, respectively). The correlation between the self-rated FRQ and the Thai-FRAT was fair (|*r*| = 0.487). When analyzing correlations within only the fracture group, we found moderately strong correlations between the score of the self-rated FRQ and the scores of the other outcome measurements (|*r*| ranging from 0.503 to 0.658). In contrast, the correlations in the non-fracture group were lower with fair correlations between the self-rated FRQ and the Thai-FRAT, TUG, and 5TSTS (|*r*| ranging from 0.300 to 0.430), and moderately strong correlation between the self-rated FRQ and the BBS (|*r*| = 0.658) (Table 3).

**Table 3:** Construct validity of the self-rated FRQ relative to other outcome measurements

Outcome measurement tool	Self-rated FRQ					
	Total (***N*** = 68)		Fracture group (***n*** = 34)		Non-fracture group (***n*** = 34)	
	***r***	***p-***value	***r***	***p-***value	***r***	***p-***value
Thai-FRAT	0.487	***< 0.001***	0.503	***0.002***	0.430	***0.011***
BBS	−0.690	***< 0.001***	− 0.658	***< 0.001***	− 0.658	***< 0.001***
TUG test	0.535	***< 0.001***	0.628	***< 0.001***	0.300	0.085
5TSTS test	0.566	***< 0.001***	0.646	***< 0.001***	0.386	***0.024***

A *p*-value< 0.05 indicates statistical significance (Spearman’s rank correlation)

*r* – Spearman’s rank correlation coefficient

*Abbreviations***:** *Thai-FRAT* Thai Falls Risk Assessment Test, *BBS* Berg Balance Scale, *TUG test* Timed get-up-and-go test, *5TSTS test* 5 times sit-to-stand test

No floor or ceiling effects were observed in any of the 5 evaluated assessment tools. Test-retest reliability of the self-rated FRQ was high with a Kappa of 1 (see Additional file 1). Internal consistency of the self-rated FRQ was excellent with a Cronbach’s alpha of 0.936.

## Discussion

The most common cause of fracture in osteoporotic populations is falling from a standing height or less, and 79% of hip fractures result from a simple fall [24]. In the United States, fall-related deaths among patients aged over 65 years increased 31% from 2007 to 2016, and falls are the leading cause of death from unintentional injuries in this population [25]. Therefore, fall risk reduction is an essential component of both primary and secondary fragility fracture prevention strategies, which are usually implemented via a multidimensional approach. Components of this strategy include calcium and vitamin D supplementation, exercise, medication review, anti-osteoporotic drugs, and fall risk assessment. Although a number of fall risk assessment tools have been developed to evaluate fall risk in older adult population, no single test can determine the risk of falls accurately. In this study, we demonstrated that the self-rated FRQ is a valid and reliable tool for assessing fall risk in older adult population with osteoporosis.

Osteoporosis is commonly diagnosed using BMD T-score or by the occurrence of a fragility fracture [26]. However, these two diagnostic subgroups are different. As shown in our study, patients in the fracture group were relatively older, had a higher Charlson comorbidity index, and had a higher number of patients who required a gait aid during ambulation. In addition, fracture patients had higher scores on the self-rated FRQ, and performed poorer on the performance-based tests. This may suggest that osteoporotic patients with a history of fragility fracture tend to be naturally more frail than those without fracture [27–29]. Fragility fracture can also adversely impact mobility, which leads to physical inactivity and further increases the risk of falls [30, 31]. Therefore, an attempt to reduce falls is of prime importance by identifying at-risk individuals, especially among those who have already sustained a fragility fracture.

Our findings showed that the self-rated FRQ demonstrated acceptable levels of validity and reliability for predicting falls in older adults with osteoporosis. The self-rated FRQ had moderately strong correlation with the performance-based tests, including BBS, TUG test, and 5TSTS test (|*r*| ranging from 0.535 to 0.690). The previous study that evaluated the validity of another Thai-translated self-rated FRQ in community-dwelling older Thai adults found only fair correlation between their self-rated FRQ and the BBS and TUG (|*r*| ranging from 0.330 to 0.499) [32]. This disparity in the degree of correlation may be attributable to differences in the study populations (community-dwelling older adults versus osteoporotic patients). When analyzing correlations between the self-rated FRQ and other fall risk assessment tests only in fracture patients, the strength of the correlations was stronger than in those who were diagnosed with osteoporosis from BMD alone. This suggests that the self-rated FRQ is more accurate in determining falls, especially among those with history of fragility fracture. It is clear that older adult patients with fragility fracture are at higher risk for falls compared to the general older adult population [4, 33]. Higher rates of subsequent falls are also frequently reported in osteoporotic patients [4, 34]. Moreover, osteoporotic patients are at greater risk for fractures than other healthy older adults due to lower bone strength [33]. Approximately 10–15% of falls in patients aged over 65 years result in fractures [35], whereas osteoporotic patients sustained a rate of fall-related fractures of 40% [36]. Therefore, the self-rated FRQ should be implemented as part of a fall risk assessment test in osteoporotic patients, especially in those with fragility fracture.

A previous meta-analysis study showed performance-based tests to be the strongest predictor of falls [18]. The fact that the self-rated FRQ was found to be moderately strongly correlated with the three performance-based tests (BBS, TUG test, and 5TSTS test) used in this study suggests that the self-rated FRQ is an assessment tool that can reliably evaluate fall risk in older adults with osteoporosis. In addition, the fact that the self-rated FRQ significantly, but only fairly significantly, correlated with the Thai-FRAT, which consists mainly of questions specifically designed to identify fall risk factors, suggests that both share similar features only in some dimensions. The self-rated FRQ also has different ability to assess the physical performance of patients. Thus, the self-rated FRQ contains items that assess both physical performance and risk of falls in older adults with osteoporosis.

Although performance-based tests have been recommended by some investigators for their clinical usefulness in determining risk of falls in older adult patients [16–20], there are some disadvantages regarding the difficulty of administering these tests in real-life clinical settings, especially outpatient settings. Some physicians reported that in addition to performance-based tests being time-consuming, they also require specific knowledge and training to perform [37, 38]. Moreover, performance-based tests might not be suitable for some older adult patients, especially those with fragility fracture who are at high risk for falls [7]. To address these problems, most questionnaires were developed to include a number of fall-related factors [18, 39]; but still, those questionnaires do not contain questions that capture distinct domains of performance in activities of daily living. In other words, the actual level of patient functional ability contributes to different degrees of fall-related injury, and the likelihood of falling hasn’t been thoroughly assessed [40]. The simplicity, safety, ease of use, and the fact that it can be used in all osteoporotic patients, including those with fragility fractures, makes the self-rated FRQ an attractive fall risk assessment in this patient population. In addition, this self-screening tool will reduce assessment time for physicians, and it will not overburden patients. Lastly and importantly, the self-rated FRQ can be conveniently completed by the patient at home or in the waiting room.

The strength of this study is that it is the first study to evaluate the validity and reliability of the self-rated FRQ in osteoporotic patients. Since fall risk is higher in older adults with osteoporosis [3, 4], our self-rated FRQ, which is a simple, practical, and easy-to-conduct test, should be incorporated into routine clinical practice to predict the risk of falls. This study also has some mentionable limitations. First, the majority of our subjects were able to ambulate without gait aid; therefore, our results may not be generalizable to other populations, such as those who require a gait aid. The validity and reliability of this self-rated FRQ could be different if performed in those with physical limitations. Second, we assessed the validity of the self-rated FRQ via comparison with 3 performance-based tests because evidence supported the use of BBS, TUG, and 5TSTS tests as fall risk assessment tests in older adult population [18]. However, other performance-based tests are available, and we are not able to recommend which ones are best for evaluating fall risk in older adult patients with osteoporosis.

## Conclusions

The self-rated FRQ is a valid and reliable tool for assessing the risk of falling in older adult patients with osteoporosis, including those with a history of fragility fracture. We also propose the use of the self-rated FRQ in the fracture liaison service to improve continuity of care in both primary and secondary prevention of fragility fractures.

## Supplementary Information

**Additional file 1:** **Supplementary table.** Reliability of the self-rated fall risk questionnaire.

## Data Availability

The data collected and analyzed in the current study are available from the corresponding author on reasonable request.

## Abbreviations

Self-rated FRQ: Self-rated Fall Risk Questionnaire
Thai-FRAT: Thai Falls Risk Assessment Test
BBS: Berg Balance Scale
TUG test: Timed get-up-and-go test
5TSTS test: 5 times sit-to-stand test
STEADI: Stop Elderly Accidents, Deaths, and Injuries
