# Supplementary material for: Validity and reliability of the self-rated fall risk questionnaire in older adults with osteoporosis
Source: BMC Musculoskelet Disord. 2020 Nov 18;21:757. doi: 10.1186/s12891-020-03788-z (PMC7677850; doi:10.1186/s12891-020-03788-z)
Supplement: Supplementary file 1 — Additional file 1: Supplementary table. Reliability of the self-rated fall risk questionnaire. [file 12891_2020_3788_MOESM1_ESM.pdf]

Supplementary table

Reliability of the self-rated fall risk questionnaire

self-rated FRQ	retest-self-rated FRQ		Test-retest reliability (Kappa statistics)	*p* value
	yes	no		
I have fallen in the past year.				
- yes	8 (100.0)	0 (0.0)	1.000	< 0.001
- no	0 (0.0)	22 (100.0)		
I use or have been advised to use a cane or walker to get around safely.				
- yes	15 (100.0)	0 (0.0)	1.000	< 0.001
- no	0 (0.0)	15 (100.0)		
Sometimes I feel unsteady when I am walking.				
- yes	17 (100.0)	0 (0.0)	1.000	< 0.001
- no	0 (0.0)	13 (100.0)		
I steady myself by holding onto furniture when walking at home.				
- yes	12 (100.0)	1 (5.6)	0.932	< 0.001
- no	0 (0.0)	17 (94.4)		
I am worried about falling.				
- yes	16 (100.0)	0 (0.0)	1.000	< 0.001
- no	0 (0.0)	14 (100.0)		
I need to push with my hands to stand up from a chair.				
- yes	16 (100.0)	0 (0.0)	1.000	< 0.001
- no	0 (0.0)	14 (100.0)		
I have some trouble stepping up onto a curb.				
- yes	16 (100.0)	0 (0.0)	1.000	< 0.001
- no	0 (0.0)	14 (100.0)		
I often have to rush to the toilet.				
- yes	14 (100.0)	3 (18.8)	0.802	< 0.001
- no	0 (0.0)	13 (81.3)		
I have lost some feeling in my feet.				
- yes	7 (100.0)	1 (4.3)	0.911	< 0.001
- no	0 (0.0)	22 (95.7)		
I take medicine that sometimes makes me feel light-headed or more tired than usual.				
- yes	2 (100.0)	1 (3.6)	0.783	< 0.001
- no	0 (0.0)	27 (96.4)		
I take medicine to help me sleep or improve my mood.				
- yes	7 (100.0)	2 (8.7)	0.831	< 0.001

self-rated FRQ	retest-self-rated FRQ		Test-retest reliability (Kappa statistics)	*p* value
	yes	no		
- no	0 (0.0)	21 (91.3)		
I often feel sad or depressed.				
- yes	5 (100.0)	0 (0.0)	1.000	< 0.001
- no	0 (0.0)	25 (100.0)		
Total score				
- yes	19 (100.0)	0 (0.0)	1.000	< 0.001
- no	0 (0.0)	11 (100.0)		

Abbreviations: *p* value = Kappa statistics

p value < 0.05 indicates statistical significance

self-rated FRQ self-rated Fall Risk Questionnaire
